# Inflexible daily behaviour is associated with the ability to control an automatic reaction in autism spectrum disorder

**DOI:** 10.1038/s41598-018-26465-7

**Published:** 2018-05-24

**Authors:** Shisei Tei, Junya Fujino, Ryu-ichiro Hashimoto, Takashi Itahashi, Haruhisa Ohta, Chieko Kanai, Manabu Kubota, Motoaki Nakamura, Nobumasa Kato, Hidehiko Takahashi

**Affiliations:** 1 Medical Institute of Developmental Disabilities Research, Showa University, 6-11-11 Kita-karasuyama, Setagaya-ku, Tokyo, Japan; 2 Department of Psychiatry, Graduate School of Medicine, Kyoto University, 54 Shogoin-Kawaracho, Sakyo-ku, Kyoto, Japan; 3 School of Human and Social Sciences, Tokyo International University, 2509 Matoba, Kawagoe, Saitama, Japan; 4 Institute of Applied Brain Sciences, Waseda University, 2-579-15 Mikajima, Tokorozawa, Saitama, Japan; 5 Department of Language Sciences, Graduate School of Humanities, Tokyo Metropolitan University, 1-1 Minami-Osawa, Hachioji-shi, Tokyo, Japan; 6 Department of Psychiatry, School of Medicine, Showa University, 6-11-11 Kita-karasuyama, Setagaya-ku, Tokyo, Japan; 7 Department of Functional Brain Imaging Research, National Institute of Radiological Sciences, National Institutes for Quantum and Radiological Science and Technology, 4-9-1 Anagawa, Inage-ku, Chiba, Japan; 8 Kanagawa Psychiatric Center, 2-5-1 Serigaya, Yokohama, Kanagawa Japan

## Abstract

Inflexible behaviours in people with autism spectrum disorder (ASD) broadly obstruct social communication. Meanwhile, flexibility implicates cognitive control to resolve socially conflicting situations; however, it remains unclear how people with ASD behave in the face of these conflicts in this respect. We used the ultimatum game (UG) and the implicit-association test (IAT) to examine goal-directed/economic flexibility, both of which involve conflict and cognitive control. In addition, we used the Detail and Flexibility Questionnaire (DFlex) to measure inflexible everyday behaviour with diminished cognitive control and attention shifting. We observed the decreased flexibility in participants with ASD (DFlex and IAT); further, their IAT scores positively correlated with DFlex. However, in the UG, contrary to our prediction, participants with ASD accepted unfair offers more frequently than TD. These results suggest that assessing the automatic/attention processing level with the IAT could be a useful approach to study behavioural flexibility among ASD compared with the UG, which might comprise multiple response strategies besides economic rationality. Overall, the severity of inflexible daily behaviours in people with ASD may be associated with a reduced flexible attitude at an automatic level, altered attention processing and decreased cognitive control.

## Introduction

Inflexible behaviours in people with autism spectrum disorder (ASD) greatly hinder social interaction^1–4^. Flexibility allows efficient and adaptive responses to changing situations in various social environments^5^. Accordingly, flexibility implicates cognitive control to achieve behavioural goal by shifting or maintaining actions depending on the situations^1,5^. Such goal-directed flexibility suppresses inappropriate impulsive emotion to resolve conflicts^6,7^. However, it remains unclear how people with ASD behave in the face of socially conflicting situations in this respect. More specifically, inflexible behaviour during various socially conflicting situations may often relate to reduced cognitive processing over automatic processing; however, this is insufficiently understood in ASD^1,8,9^. Therefore, characterising ASD’s goal-directed flexibility by different measures would be informative.

**Figure 1:**
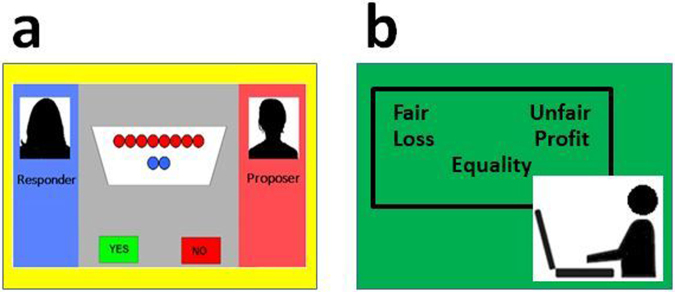
Overview of experimental measures for evaluating behavioural flexibility. **(a)** Ultimatum game: In this task, the proposer offered to split a sum of ten coins with the participant (i.e. responder). The participant was told that if he/she accepted the offer, both the proposer and the responder would be paid accordingly; however, if the participant rejected the offer, neither the proposer nor the responder would receive any payment. Twenty-five monetary offers were proposed to the participants, each containing red and blue coins indicating the share for the proposer (red coins) and for the responder/participants (blue coins). **(b)** Implicit-association test (IAT): IAT comprises a series of response time tasks that require participants to classify word stimuli that appear at the lower portion of a computer screen into corresponding categories and paired attributes appearing in the upper left or right. In this study, we applied the IAT concerning moral trade-offs to maximise social welfare (i.e. profit/loss vs. fairness/unfairness attributes).

Goal-directed flexibility has been assessed by different approaches to examine individuals’ shifting of perspective and decision-rule in various social contexts; these approaches could be more relevant to daily situations^1^. For example, individuals’ flexible behaviour is investigated as economic^5^ and moral^10^ flexibilities, in addition to perceptual/cognitive flexibility^11^. Thus, one aspect of goal-directed flexible behaviour can be examined through monetary games from an economic rationality perspective. Namely, the goal-directed/economic aspect of flexibility can be assessed by the ultimatum game (UG; Fig. 1a)^5,12^. In the UG, the proposer (the first player) proposes how to divide a sum of money between the two players and the responder (the second player; experimental participant) can either accept or reject this proposal. If the responder rejects it, neither player receives anything; however, if the responder accepts it, the money is split according to the proposal^13^. In this UG, accepting an unfair proposal can represent goal-directed flexibility^6^ that requires cognitive control^14^ to resolve the conflict between obtaining practical benefits and emotional distress^5,15^. That is, those with more goal-directed flexibility may be better at prioritising economic gains over inequality aversion that balances competition and cooperation with other individuals^12^. Although we are intrinsically averse to unfairness/inequality during social communication^16^, successfully navigating social life often requires to establish rational goals via cognitive control^17–19^. Such cognitive control would support maximising the profit/welfare of oneself as well as others by flexibly accepting unfair proposals^6,13,17,20^.

A previous study has shown that individuals with ASD indeed rejected unfair offer^21^ relatively more often than those with typical development (TD) in the mini-UG. This result is relatively comparable with other psychiatric diseases with flexibility deficit^22^; for example, compared with controls, alcohol-dependent individuals rejected unfair offers more frequently^23^. However, other studies reported that participants with ASD accepted unfair offers more frequently in the UG, related to the difficulty in shifting of decision strategy^24^, and showed a reduced tendency to reciprocate other’s offer^25^ in a rigid rule-oriented manner. Besides, a recent UG study in ASD (enrolled as proposer) reported that participants distributed more money than TD; however, they also behaved rather consistently irrespective of backgrounds condition where social and non-social stimuli were displayed^26^. Thus, although people with ASD made somewhat similar level of cooperative decisions as those with TD^27^, they also exhibited an atypical response, which might be associated with reduced cognitive control^28^, altered attentional/emotional processing^1^ and rigid or restricted/stereotyped attitude^29^. Here, an additional approach on these atypical responses could facilitate investigating ASD’s inflexible behaviour.

Thus, we conducted the implicit-association test (IAT)^30^ to further investigate goal-directed flexibility with an intention to assess preferences for goal-directed/economic flexibility and the cognitive control of automatic responses^31,32^. IAT is widely used and is a validated approach to study individual differences in automatic/implicit aspects of personality and social cognition^33^. IAT comprises a series of tasks that require participants to classify word stimuli into corresponding categories based on the paired attributes (Fig. 1b). The underlying assumption of IAT is that past learning experiences could be represented by the facilitation of the information processing of associated concepts, as measured by the response time^30^. Thus, automatic attitudes are exhibited as the duration of button-press responses, controlled by participants’ implicit evaluations^30^. In particular, response times are expected to be shorter when paired target category and attribute labels match an individual’s automatic associations. Meanwhile, response times are expected to be longer when paired target category and attribute labels contradict automatic associations of which participants are unaware (i.e. higher IAT effect or D score^30^; please see Supplementary Information). Among people with ASD, studies have reported an increased trend in latency-based IAT effects^34^, which might represent their use of rule-based, stereotype knowledge^29^. This result might also be affected by their atypical attentional/emotional processing and cognitive control^35^.

Based on the abovementioned information, we applied IAT that was used in our previous flexibility study^20^. To assess individuals’ preference for goal-directed flexible attitude via cognitive control^26,27^, attributes of this IAT concerned moral trade-offs to maximise social welfare. Namely, we used profit/loss vs. fairness/unfairness attributes: Individuals with greater flexibility^36,37^ would be expected to be more easily diverted from moral perceptions as assessed by the response time of the button pressed via increased cognitive control^26,27^ (i.e. higher tolerance for unfairness and rule-based attitudes). In other words, those with lower flexibility would tend to show a much longer response time during vocabulary categorisation in the incongruent condition of IAT, where unfairness was paired with a positive attribute (financial gain), as compared to categorisation in the congruent condition, where unfairness was paired with a negative attribute (financial loss)^20^.

This study aimed for a better understanding of ASD’s inflexible behaviours in light of cognitive control. We examined participants’ level of goal-directed flexibility by applying the UG and IAT, both of which involve cognitive control to deal with decision-conflict. In addition, we used the Detail and Flexibility Questionnaire (DFlex)^38^ to measure inflexible everyday behaviour with diminished cognitive control and set-shifting (i.e. attention to detail)^39,40^. This was because these rigid and stereotyped daily attitudes can be also linked with atypical automatic/implicit reaction in ASD^1^. We hypothesised that ASD would show a decrease in both daily flexible behaviour (as assessed via the DFlex) and goal-directed flexibility (as assessed via UG and IAT) compared with TD. In addition, on the basis of previous studies^41,42^, we predicted a correlation between the level of inflexible daily behaviour and the severity of diminished goal-directed flexibility in ASD.

## Methods

### Participants

A total of 49 volunteers participated in this study (age: 20–45 years). Twenty-five ASD participants were matched to 24 TD participants on age, intelligence quotient (IQ) and gender (Table 1). ASD participants were recruited from a database of volunteers who had received a clinical diagnosis of ASD in outpatient units of the Showa University Karasuyama Hospital in Tokyo, Japan. The diagnostic procedure used to identify ASD was the same as that used in our previous studies^43–45^. At least three experienced psychiatrists and a clinical psychologist assessed and confirmed the participants using the criteria of the Diagnostic and Statistical Manual of Mental Disorders Fourth Edition, Text Revision (DSM-IV-TR). The assessment consisted of participant interviews about developmental history, present illness and life/family history. Patients were also asked to bring suitable informants who had known them in early childhood. This process required approximately 3 hours. A diagnosis of ASD was made only when there was a consensus between the psychiatrists and clinical psychologist. At the time of testing, an experienced psychiatrist evaluated psychiatric comorbidity using the Structured Clinical Interview for DSM-IV Axis I Disorders (SCID). No ASD participants satisfied the diagnostic criteria for substance use disorder, bipolar disorder or schizophrenia. TD participants were recruited through advertisements and acquaintances. They did not meet the criteria for any psychiatric disorders according to SCID performed by an experienced psychiatrist. No participants (ASD or TD participants) had any history of head trauma, serious medical or surgical illness. In the ASD group, a total of 12 participants were administered the following psychotropic drugs: anxiolytics (n = 4), antidepressants (n = 6), antipsychotics (n = 4), antiepileptics (n = 3), sleep-inducing drugs (n = 6) and other psychotropic drugs (n = 3). The participants overlapped partially with those included in our previous study^43^. In this study, we obtained the results by theoretically and methodologically distinct analyses of the previous dataset (please see Supplementary Methods for details).

**Table 1:** Demographic data of participants.

	ASD group (*n* = 25) mean ± SD	TD group (*n* = 24) mean ± SD	*p*-value
Age (years)	28.9 ± 4.3	26.5 ± 6.7	0.09
Females/Males	2/23	2/22	0.97^a^
IQ	105.2 ± 11.3	106.2 ± 8.2	0.53
Current smokers/non-smoker	2/23	3/21	0.60^a^
AQ	34.0 ± 5.6	15.8 ± 6.7	<0.01

Abbreviations: ASD = autism spectrum disorder; AQ = Autism-Spectrum Quotient; IQ = intelligence quotient; SD = standard deviation; TD = typical development.

^a^Chi-square test.

The IQ scores of all ASD participants had been evaluated before the study using either the Wechsler Adult Intelligence Scale-Third Edition (WAIS-III) or the WAIS-Revised (WAIS-R). All ASD participants scored above 80 and were considered to be high functioning. Although there are several minor changes in WAIS-III from WAIS-R (e.g. more items), the number of core items remained largely unchanged. Therefore, we considered that the WAIS-R and WAIS-III were essentially the same with regard to measuring the full-scale IQ score of individuals with ASD. The IQ scores of the TD participants were estimated using a Japanese version of the National Adult Reading Test (JART), based on the previous findings that JART successfully predicted the full-scale IQ score in the healthy population^46^. JART is equivalent to the National Adult Reading Test (NART; comprising a list of 50 words printed in the order of increasing difficulty) and comprises random Japanese words, all of which are complicated Kanji (ideographic script). In addition, JART has proven good validity for evaluating the IQ level; and the estimated IQ score by JART is reasonably comparable with that of WAIS^47^. This study was approved by the Committee on Medical Ethics of Kyoto University and the Institutional Review Board of Showa University Karasuyama Hospital^43^ and was carried out in accordance with the Code of Ethics of the World Medical Association. After providing a complete description of the study, written informed consent was obtained from all participants, who were compensated for their participation.

### Psychological measure

We assessed participants’ autistic traits using the Japanese version of the 50-item Autism-Spectrum Quotient (AQ) test^48^, which comprises items concerning social and non-social aspects of behaviour and cognition (e.g. ‘I am fascinated by numbers’ and ‘I am often the last to understand the point of a joke’). AQ has been widely used to quantify autistic traits in research and clinical practice^43^. A high AQ score implies strong autistic traits. In addition, we administered DFlex to examine participants’ inflexible daily behaviour. DFlex has been validated for self-report use in adults with eating disorders^38^; it has also been applied to evaluate reduced flexibility regarding autistic traits^49–51^. DFlex comprises 24 items, each rated on a six-point Likert scale (with anchors strongly agree and strongly disagree) with statements comprising the management of unexpected challenges, changes of daily routines and adapting their plans to accommodate others. Moreover, DFlex comprises a cognitive rigidity subscale (12 items; e.g. ‘I dislike change’ and ‘I get upset if other people disturb my plans for the day by being late’) and attention to detail subscale (12 items; e.g. ‘I sometimes bore others as I go on to an excess about somethings’ and ‘I find it difficult to remember the story line in films, plays or books, but can remember specific scenes in great detail’). Higher sum scores on this scale imply higher cognitive rigidity and more attention to detail (i.e. lower flexibility)^38^. Furthermore, DFlex shows high internal reliability (Cronbach’s alpha was 0.91 and 0.88 for cognitive rigidity subscale and attention to detail subscale, respectively) and construct validity^38^.

### Behavioural measures

#### Ultimatum game

The UG is a well-established measure that manipulates conflicts between financial and fairness interests, thereby enabling the observation of behavioural flexibility (Fig. 1a; see Supplementary Information for details)^5,6^. Our experimental participants played the role of responders against a proposer. In this task, the proposer offered to split a sum of ten coins (i.e. 100 Japanese yen, with one coin corresponding to 10 Japanese yen, or approximately 0.10 US dollars) with our experimental participant (i.e. the responder). The participant was told that if he/she accepted the offer, both the proposer and the responder would be paid accordingly, but if the participant rejected the offer, neither the proposer nor the responder would receive any payment. Following the criteria from used in the previous study^13^, we also established five types of offers. That is, the proposer offered the responder 50, 40, 30, 20 or 10 yen. To this end, participants played 25 trials. To this end, fair offers (i.e. proposer’s 50 and 40 yen offers which is 40% of total offers: 10 offers) and unfair offers (60% of total offers: 15 offers) were presented randomly. Subsequently, we calculated participants’ acceptance rates. As per previous studies, to avoid learning and reputation effects, as well as to build a realistic setting, each trial was performed with a new proposer (i.e. a gender matched anonymous proposer with their first name stated in each trial). Participants were informed that at the end of the task, the computer would randomly select three trials and compute their earnings, and these payments would be added to their final compensation. In reality, all participants received the maximum possible earning. In addition, we also assessed participants’ response time of the button press during the UG. Notably, the justification might still be required to apply the UG among individuals with ASD, which is attributed to their fairness recognition^52^ and altered self-other reciprocity^53^.

#### Implicit-association test

To study goal-directed flexibility, we also conducted the IAT^30^. This was intended to measure preferences for goal-directed/economic rationality^20^ and cognitive control of automatic responses^31,32^. IAT is a widely validated method to study individual differences in automatic aspects of personality and social cognition^33^. The IAT comprises a series of tasks that require participants to classify word stimuli into corresponding categories on the basis of the paired attributes appearing in the upper left or right of the screen (Fig. 1b). The basic assumption of the IAT is that past learning experiences can be represented by the facilitation of information processing of associated concepts as measured by the rate of processing and the response time^30^. Thus, automatic attitudes are exhibited by the duration of button press responses, controlled by participants’ implicitly activated evaluations^30^. More specifically, response times are expected to be shorter when paired target category and attribute labels match an individual’s automatic associations. On the other hand, response times are expected to be longer when paired target category and attribute labels contradict automatic associations of which participants are unaware (i.e. IAT effect or greater D score; see Supplementary Information for the details)^30^. To this end, we applied IAT, which was used in our previous flexibility study^20^, to assess individuals’ preference for goal-directed flexible attitude via cognitive control. This IAT concerned moral trade-offs to maximise social welfare; namely, we used the profit/loss vs. fairness/unfairness attributes. Individuals with greater flexibility^36,37^ would be expected to more easily divert from moral perceptions as assessed by the response time of the button press by cognitive control^26,27^ (i.e. higher tolerance for unfairness and rule-based attitudes). This is because flexible individuals who have developed sophisticated strategies would form a relatively weak automatic association between unfairness and negative valence^20^.

### Procedure

We obtained informed consent from participants after they understood the overview of our experiment. The experiment was designed to carry out in the following order: IAT, UG and psychological questionnaires, followed by SCID.

### Statistical analyses

In this study, the normality of the distribution of each variable was evaluated using the Shapiro-Wilk test. Results showed that some data deviated from normality (*p* < 0.05). Subsequently, we conducted the Brunner–Munzel test^54,55^ to compare group differences and Spearman’s rank test for correlation analyses implemented in the lawstat package within the statistical software R^56^ (https://www.r-project.org). The Brunner–Munzel test is a non-parametric group comparison that corresponds to independent sample *t*-tests. This test has no assumptions for the homogeneity of variance, and assesses whether the medians of two sample distributions are equivalent^54^. Specifically, we examined whether scores on the DFlex, UG and IAT differed statistically between the ASD and TD groups. Subsequently, we assessed whether these measures correlated within ASD participants. Furthermore, for the UG, we performed the analysis of variance (ANOVA) using the bwtrim function in the software R^56^, which returns the test statistic value of Q that is approximately F-distributed^57^. This function returns neither degrees of freedom nor effect sizes^57^. In the current study, the threshold for the statistical significance was set at *p* < 0.05 (two-tailed).

## Results

Twenty-five ASD and 24 TD participants took part in this study and were included in statistical analyses. As shown in Table 1, there were no significant differences in average age, gender, IQ, handedness or current smoking status between the groups (we asked participants’ current smoking status because smoking can influence reward-related decision-making^58^). Meanwhile, the AQ score was significantly higher in the ASD group. Regarding the UG, consistent with the previous studies^13^, participants in both groups were indeed more likely to accept fair offers than unfair offers (*p* < 0.001 for both ASD and TD), which confirms that both groups understood the rule and conditions in the UG and showed tendency to choose the fair offers more frequently. In particular, the mean acceptance rates for unfair and fair offers among the ASD group were 0.43 ± 0.41 (mean ± SD) and 0.91 ± 0.18 respectively, whereas, those among the TD group were 0.20 ± 0.40 and 0.80 ± 0.24, respectively. However, contrary to our prediction, there was a greater acceptance rate of unfair offers among the ASD group than in the TD group (Brunner-Munzel test statistic = 3.33, *p* = 0.002), which could imply greater economic flexibility. Namely, the ASD group accepted unfair offers more than twice as often as the TD group as stated above (ASD: 0.43 ± 0.41; TD: 0.20 ± 0.40; Table 2). Furthermore, we conducted 2 × 2 ANOVA where factor 1 was Fairness condition (fair/unfair) and factor 2 was the Group status (ASD/TD). The results showed the significant main effect of Fairness (*Q* = 67.52; *p* < 0.001), suggesting that participants tended to accept fair offers more frequently compared with unfair offers, regardless of groups (acceptance rate: 0.86 and 0.32 for fair and unfair, respectively). Furthermore, we also observed the main effect of Group (*Q* = 5.22; *p* = 0.030), suggesting that overall, participants with ASD accepted offers more frequently than TD regardless of fairness offer types (mean acceptance rate: 0.67 and 0.50, respectively). Moreover, Fairness × Group interaction did not reach the significant threshold (*Q* = 0.92; *p* = 0.346), suggesting that ASD group’s decisions were only relatively less condition-depended (fair/unfair) compared with the TD group. Regarding the reaction time of the button press in the UG, we observed a statistically significant difference between the two groups during the unfair offers (statistic = 2.21; *p* = 0.032), indicating that ASD groups spent longer time than the control (TD) group during unfair offers. Meanwhile, the result of fair offers between the two groups was not significant (statistic = 0.39; *p* = 0.698). Specifically, the mean reaction time for unfair and fair offers among the ASD group were 1.47 ± 0.77 (mean ± SD) and 1.23 ± 0.51 respectively, whereas those among the TD group were 1.10 ± 0.45 and 1.22 ± 0.61, respectively.

**Table 2:** Behavioural characteristics of participants.

	ASD group (*n = *25) mean ± SD	TD group *(n* = 24) mean ± SD	*p*-value
UG	0.43 ± 0.41	0.20 ± 0.40	0.002**
IAT	0.86 ± 0.28	0.67 ± 0.39	0.042*
DFlex	98.60 ± 13.89	76.00 ± 13.16	<0.001**

Abbreviations: ASD = autism spectrum disorder; DFlex = Detail and Flexibility Questionnaire; IAT = implicit-association test; SD = standard deviation; TD = typical development; UG = ultimatum game (acceptance rate of unfair offers). *p* < 0.05*; *p* < 0.01**.

**Figure 2:**
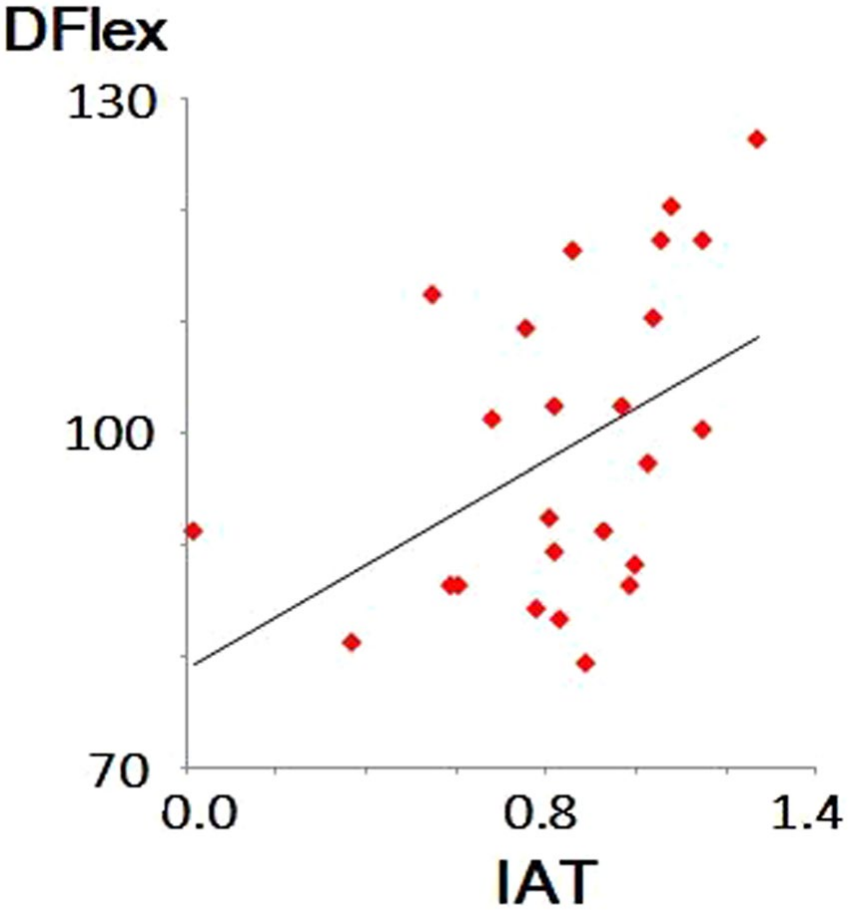
Scatter plot of scores on the implicit-association test (IAT) and Detail and Flexibility Questionnaire (DFlex) among participants with ASD. DFlex scores positively correlated with the D scores of our IAT (*rho* = 0.51, *p* = 0.009). This IAT concerned moral trade-offs to maximise social welfare. Namely, we used profit/loss vs. fairness/unfairness attributes; please see Methods for more details).

Regarding IAT, there was a reliable IAT effect in both the groups. The D scores of our IAT in the ASD and TD groups were 0.86 ± 0.28 and 0.67 ± 0.39, respectively. These positive D scores implied that both ASD and TD groups responded more slowly in the incongruent IAT phase than in the congruent IAT phase (i.e. IAT effect)^59^, representing a potentially stronger automatic association between unfairness and negative valence^16^. Furthermore, there was a statistically greater IAT effect in the ASD than in the TD (i.e. higher D score representing lower flexibility; statistic = 2.09, *p* = 0.042; Table 2). Moreover, as expected, we observed a distinctive increase in DFlex scores (inflexible daily behaviours) in the ASD group as compared to the TD group (Brunner–Munzel test statistic = 8.37, *p* < 0.001; Table 2). In addition, this DFlex scores positively correlated with the D scores of our IAT among the ASD group (*rho* = 0.51, *p* = 0.009; Fig. 2). However, correlations between scores on the DFlex and UG (*rho* = 0.18, *p* = 0.394) and on the UG and IAT (*rho* = −0.05, *p* = 0.804) were non-significant.

## Discussion

This study aimed for a better understanding of the inflexible behaviours in ASD. In line with the previous studies^1,49^, we observed the decreased flexibility in ASD. This was characterised by goal-directed/economic flexibility and inflexible everyday behaviour, as measured by the IAT and DFlex, respectively. Moreover, IAT scores positively correlated with DFlex scores. One possible interpretation for this association could be that the severity of inflexible everyday behaviour may relate to the decreased cognitive control of automatic responses and reduced preference for goal-directed/economic attitude in ASD; and that the shifting of decision-rule may play a crucial role^1,2^. Further study is required to explore whether such a diminished flexible attitude at the automatic level is an important mechanism representing inflexible daily behaviours. However, in the UG, contrary to our prediction, our ASD participants showed an increased acceptance rate of unfair offers, and this UG score did not statistically correlate with the IAT or DFlex scores. These results suggest that assessing the level of the automatic/attention processing could be a useful approach to investigate the behavioural flexibility among individuals with ASD compared with the UG, which might comprise multiple response strategies besides economic rationality.

The observed association between the IAT and DFlex scores may indicate that the inflexibility of people with ASD could be affected by altered attention processing^3,60^. This could be because the higher DFlex scores observed in the ASD participants may have represented their difficulty in cognitive set-shifting^11^ and their tendency to pay attention to detail^38,49^. Additionally, the greater IAT effect could also have arisen due to reduced cognitive regulation for controlling automatic responses^31,32,36,37^, which involves intensive emotional disengagement from intrinsic attribute patterns^61^. Thus, it could be possible that the IAT-DFlex association may further suggest that those with a weaker ability to attentively control automatic reactions show more inflexible and stereotyped everyday behaviours. Indeed, reduced attention processing and inflexible behaviours could be linked via cognitive control^3^. However, additional research should be conducted to elucidate this issue because these associations remains unclear^1,62,63^.

Regarding the UG, we performed ANOVA (Fairness × Group) and observed a main effect of Group, suggesting that overall, participants with ASD accepted offers more frequently than TD regardless of fairness offer types. This might imply a qualitative difference in responding to the UG between the ASD and TD groups. In particular, although both ASD and TD might sustain an innate desire for their material possessions, their decision-making during the UG may not entirely align with the economic model of rational pursuit of self-interest^64^, which perhaps could be associated with ASDs’ atypical egocentrism (e.g. restricted or stereotyped behaviour) and self-other reciprocity^52,53^, as well as altered reasoning and emotional processing^8,65^. Thus, it is possible that the response in the UG among ASD might have also measured some other factors (including the above) rather than a sole flexibility.

Several limitations of this study should be noted, and thus, our results must be interpreted with caution. First, our participants included only high-functioning individuals with ASD. Future studies should include individuals with a range of IQs, age and genders. In this regard, our findings also require replication in larger samples and across different autism subtypes^1^. Second, we performed the correlation analyses for ASD group (*N* = 25), which is an underpowered analysis^66^ and warrants further justification in the future studies. Third, the validity of applying the UG in the ASD population should be investigated. It is imperative to further assess the flexibility regarding the fairness-preference and perspective-taking (emotional understanding of others) as these emotional processing could be altered in ASD^1^. Likewise, it is also essential to investigate the potential presence of a gap between the level of perceived inequality aversion and rule-based fairness preference towards others in the ASD population^52,67^. In this relation, a more robust validation of DFlex in people with ASD is warranted, although DFlex has been applied in several studies investigating autism. Fourth, IAT studies among people with ASD are rather inconclusive, whereas an increased trend was reported in latency-based IAT effects^34^ to use rule-based, stereotype knowledge^29^. However, other studies reported decreased IAT effects in ASD compared with TD, which might represent reduced social habituation of stereotype attitude^68^. Although both of these might be partly associated with atypical emotional and cognitive controls^35,69^, this issue warrants further assessment. Fifth, we did not counterbalance the task orders across participants (e.g. IAT and UG), which could have substantially affected the outcome, i.e. the initial completion of the economic game might alter responding of the subsequent psychological questionnaires^70^. Conversely, the initial completion of psychological questionnaires could also make particular preferences more salient that can alter the results of subsequent behavioural task (e.g. economic game). Sixth, the outcomes of our study could have been partially driven by a medication effect, as nearly half of our participants with ASD were taking psychotropic medication. For instance, serotoninergic and dopaminergic agents have been suggested to affect flexible decision-making^71^ as well as social/cooperative behaviour^17^. Finally, studies on the stereotyped behaviour characteristics of ASD have been inconclusive, possibly due to differing experimental approaches^29,34,68^. Thus, additional studies are required to take these issues more into account.

In this study, we used two different approaches to investigate goal-directed flexibility in the same patient population. Consequently, we showed that the severity of inflexible daily behaviours in ASD could be associated with reduced flexible preferences at the automatic level, altered attention processing and decreased cognitive control. Continued research pertaining to these may provide additional clues regarding mechanisms underlying behavioural inflexibility in ASD.

## Electronic supplementary material

Supplementary Methods
