# Supplementary material for: Inflexible daily behaviour is associated with the ability to control an automatic reaction in autism spectrum disorder
Source: Sci Rep. 2018 May 24;8:8082. doi: 10.1038/s41598-018-26465-7 (PMC5967343; doi:10.1038/s41598-018-26465-7)
Supplement: Supplementary file 1 — Supplementary Methods [file 41598_2018_26465_MOESM1_ESM.docx]

**Supplementary Information**

**Title**: Inflexible daily behaviour is associated with the ability to control an automatic reaction in autism spectrum disorder

**Authors:** Shisei Tei^1,2,3,4^, Junya Fujino^1,2^, Ryu-ichiro Hashimoto^1,5^, Takashi Itahashi^1^, Haruhisa Ohta^1,6^, Chieko Kanai^1^, Manabu Kubota^1,2,7^, Motoaki Nakamura^1,8^, Nobumasa Kato^1^, Hidehiko Takahashi^1,2*^

**Affiliations**

^1^Medical Institute of Developmental Disabilities Research, Showa University, 6-11-11 Kita-karasuyama, Setagaya-ku, Tokyo, Japan

^2^Department of Psychiatry, Graduate School of Medicine, Kyoto University, 54 Shogoin-Kawaracho, Sakyo-ku, Kyoto, Japan

^3^School of Human and Social Sciences, Tokyo International University, 2509 Matoba, Kawagoe, Saitama, Japan

^4^Institute of Applied Brain Sciences, Waseda University, 2-579-15 Mikajima, Tokorozawa, Saitama, Japan

^5^Department of Language Sciences, Graduate School of Humanities, Tokyo Metropolitan University, 1-1 Minami-Osawa, Hachioji-shi, Tokyo, Japan.

^6^Department of Psychiatry, School of Medicine, Showa University, 6-11-11 Kita-karasuyama, Setagaya-ku, Tokyo, Japan

^7^Department of Functional Brain Imaging Research, National Institute of Radiological Sciences, National Institutes for Quantum and Radiological Science and Technology, 4-9-1 Anagawa, Inage-ku, Chiba, Japan

^8^Kanagawa Psychiatric Center, 2-5-1 Serigaya, Yokohama, Kanagawa, Japan

***Corresponding author:** Hidehiko Takahashi

Department of Psychiatry, Kyoto University Graduate School of Medicine, Sakyo-ku, Kyoto 606-8507, Japan.

E-mail: [hidehiko@kuhp.kyoto-u.ac.jp](mailto:hidehiko@kuhp.kyoto-u.ac.jp)

**Supplementary Methods**

**Participants**

In this study, we enrolled 49 volunteers (age: 20–45 years). Twenty-five ASD participants were matched to 24 TD participants based on age, intelligence quotient (IQ) and gender. We recruited participants with ASD from a database of volunteers who had received a clinical diagnosis of ASD in outpatient units of the Showa University Karasuyama Hospital (Tokyo, Japan). TD participants were enrolled through advertisements and acquaintances. These participants partially overlapped with those enrolled in our previous study on ambiguity and risk[^1^](#_ENREF_1) (27 participants with ASD and 27 healthy controls). Thus, our previous study comprised 92.6% and 88.9% of the ASD and TD samples, respectively, of the current study. However, no overlap was present in the behavioural tasks in these studies. In addition, we obtained results in the current study using theoretically and methodologically distinct analyses of this previous dataset.

In the current study, no participant with ASD satisfied the diagnostic criteria for substance use disorder, bipolar disorder or schizophrenia. Regarding other current comorbidities, of 25 participants with ASD, five were diagnosed with anxiety disorder, and three with depression. Moreover, one participant was diagnosed with sleeping disorder and one with dyslexia (all were outpatients).

**Ultimatum game**

In advance of the experimental session, participants practiced the task until they demonstrated full understanding. As per previous studies, to avoid learning and reputation effects, as well as to build a realistic setting, each trial was performed with a new proposer (i.e. a different age and gender matched anonymous proposer with their first name stated in each trial). That is, participants were told a cover story that they would be playing the role of responder with volunteers who had submitted their monetary offers in advance. Participants were also informed that at the end of the task, the computer would randomly select three trials and compute their earnings, and these payments would be added to their final compensation. In reality, all participants received the maximum possible earning.

**Implicit-association test**

We applied the IAT was used in our previous flexibility study[^2^](#_ENREF_2) to assess individuals’ preference for goal-directed flexible attitude via cognitive control[^26^](#_ENREF_26)^,^[^27^](#_ENREF_27). This IAT concerned moral trade-offs to maximise social welfare; namely, using gain/loss vs. fairness/unfairness attributes. Individuals with greater flexibility[^3^](#_ENREF_3)^,^[^4^](#_ENREF_4) would more easily divert from moral perceptions as assessed by the response time of the button press by cognitive control[^26^](#_ENREF_26)^,^[^27^](#_ENREF_27) (i.e. higher tolerance for unfairness and rule-based attitudes). Because flexible individuals who have developed sophisticated strategies would form a relatively weak automatic association between unfairness and negative valence[^2^](#_ENREF_2).

Based on the above, four items were selected as the to-be-sorted stimuli for each concept. Stimuli from the fairness and unfairness categories, respectively, were: *honesty, equality, neutrality* and *conscientiousness* and *deviation, difference, irregularity* and *violation*. Stimuli from the gain and loss categories, respectively, were: *acquirement, benefit, positive* and *winning* and *debt, waste, negative* and *cost*. The present IAT comprised seven blocks according to standard procedures[^5^](#_ENREF_5): (Block 1) 20 practice trials with target categories only; (Block 2) 20 practice trials with attribute categories only; (Block 3) 20 practice trials for a congruent block with both target and attribute categories; (Block 4) 40 test trials for a congruent block with both target and attribute categories; (Block 5) 20 practice trials with target categories only in reversed positions; (Block 6) 20 practice trials for an incongruent block with both reversed target categories and the attribute categories and (Block 7) 40 test trials for an incongruent block with both reversed target categories and attribute categories. At the beginning of the task, participants were told that they would be making a series of category judgements. In each trial, a stimulus word was displayed in the centre of a computer screen. Category labels were displayed on the left and right sides of the window. Participants used the letter ‘A’ on the left side and the letter ‘L’ on the right side of the keyboard for their responses. They were also instructed to be as accurate as possible while simultaneously responding as quickly as possible. An incorrect response led to a red ‘X’ as feedback. An inter-trial interval of 300 ms was used. The IAT was conducted using E-Prime software (Psychology Software Tools, Inc., Pittsburgh, PA, USA).

The IAT effect was computed by Greenwald’s D score, based on an improved scoring algorithm[^6^](#_ENREF_6)^,^[^7^](#_ENREF_7). No participants showed latencies less than 300 ms in more than 10% of trials. Trials with latencies greater than 10000 ms and less than 400 ms were deleted. Each error latency was replaced with the sum of: 1) the mean latencies of correct responses within the same block and 2) 600 ms, according to the scoring criteria [^6^](#_ENREF_6). The mean latency in Block 3 trials was subtracted from that of Block 6. Similarly, the mean latency in Block 4 trials was subtracted from that of Block 7. Then, each difference score was divided by its associated trial standard deviation of response latencies. Finally, the D score was computed as the average of these two scores (i.e. a greater IAT effect represented a lesser level of implicit flexibility).

**References for the Supplementary Information**

1. Fujino, J.*, et al.* Attitudes toward risk and ambiguity in patients with autism spectrum disorder. *Mol Autism* **8**, 45 (2017).

2. Fujino, J.*, et al.* Role of spontaneous brain activity in explicit and implicit aspects of cognitive flexibility under socially conflicting situations: a resting-state fMRI study using fractional amplitude of low-frequency fluctuations. *Neuroscience* (2017).

3. Klauer, K.C., Schmitz, F., Teige-Mocigemba, S. & Voss, A. Understanding the role of executive control in the implicit association test: why flexible people have small IAT effects. *Q J Exp Psychol (Hove)* **63**, 595-619 (2010).

4. Miyake, A. & Friedman, N.P. The Nature and Organization of Individual Differences in Executive Functions: Four General Conclusions. *Curr Dir Psychol Sci* **21**, 8-14 (2012).

5. Greenwald, A.G., McGhee, D.E. & Schwartz, J.L. Measuring individual differences in implicit cognition: the implicit association test. *J Pers Soc Psychol* **74**, 1464-1480 (1998).

6. Greenwald, A.G., Nosek, B.A. & Banaji, M.R. Understanding and using the implicit association test: I. An improved scoring algorithm. *J Pers Soc Psychol* **85**, 197-216 (2003).

7. Lane, K.A., Banaji, M.R., Nosek, B.A., and Greenwald, A.G.,. Understanding and using the implicit association test: IV. *Implicit. Measures of Attitudes*, 59-102 (2007).
